# Facing Foodborne Pathogen Biofilms with Green Antimicrobial Agents: One Health Approach

**DOI:** 10.3390/molecules30081682

**Published:** 2025-04-09

**Authors:** Ana Karina Kao Godinez, Claudia Villicaña, José Basilio Heredia, José Benigno Valdez-Torres, Maria Muy-Rangel, Josefina León-Félix

**Affiliations:** 1 Centro de Investigación y Desarrollo, A.C., Culiacán 80110, Sinaloa, Mexico; akao221@estudiantes.ciad.mx (A.K.K.G.); bheredia@ciad.mx (J.B.H.); jvaldez@ciad.mx (J.B.V.-T.); mdmuy@ciad.mx (M.M.-R.); 2 SECIHTI-Centro de Investigación en Alimentación y Desarrollo, A.C., Culiacán 80110, Sinaloa, Mexico; maria.villicana@ciad.mx

**Keywords:** biofilms, green antimicrobials, foodborne pathogens, essential oils, bacteriophages

## Abstract

Food safety is a significant global and local concern due to the threat of foodborne pathogens to public health and food security. Bacterial biofilms are communities of bacteria adhered to surfaces and represent a persistent contamination source in food environments. Their resistance to conventional antimicrobials exacerbates the challenge of eradication, driving the search for alternative strategies to control biofilms. Unconventional or “green” antimicrobial agents have emerged as promising solutions due to their sustainability and effectiveness. These agents include bacteriophages, phage-derived enzymes, plant extracts, and combinations of natural antimicrobials, which offer novel mechanisms for targeting biofilms. This approach aligns with the “One Health” concept, which underscores the interconnectedness of human, animal, and environmental health and advocates for integrated strategies to address public health challenges. Employing unconventional antimicrobial agents to manage bacterial biofilms can enhance food safety, protect public health, and reduce environmental impacts by decreasing reliance on conventional antimicrobials and mitigating antimicrobial resistance. This review explores the use of unconventional antimicrobials to combat foodborne pathogen biofilms, highlighting their mechanisms of action, antibiofilm activities, and the challenges associated with their application in food safety. By addressing these issues from a “One Health” perspective, we aim to demonstrate how such strategies can promote sustainable food safety, improve public health outcomes, and support environmental health, ultimately fostering a more integrated approach to combating foodborne pathogen biofilms.

## 1. Introduction

### 1.1. The Importance of Food Safety and the Challenge of Bacterial Biofilms

Safe food is crucial for saving lives. Since humans began to produce their food, foodborne diseases have been a major cause of morbidity and increased mortality, imposing a substantial burden on all societies worldwide. According to estimates from the World Health Organization [1], more than 600 million people worldwide suffer from over 250 known and numerous unknown diseases, resulting in 420,000 annual deaths. In the USA, microorganisms linked to biofilms are responsible for 65% of microbial and 80% of chronic infections, including serious foodborne illnesses [2]. Over the past three decades, biofilm-associated diseases have also increased the annual economic burden. Therefore, it is crucial to establish practical approaches for eliminating the specific food exposures leading to human foodborne illness [3].

The rates of antimicrobial-resistant bacteria causing serious and life-threatening infections are rapidly rising [4]. The use of sub-therapeutic doses of antibiotics in food-producing animals as growth promoters and in therapeutic doses for controlling and treating infectious diseases has contributed to the development of antimicrobial-resistant microorganisms. Transmission of resistant bacteria from food-producing animals to humans through direct contact, handling, or consumption substantially threatens human health [5]

In the US, more than two million infections with antibiotic-resistant bacteria occur each year, resulting in USD 20 billion in economic losses. Notably, foodborne illnesses caused by *Campylobacter*, *Salmonella*, *E. coli* O157, *Listeria monocytogenes*, *Staphylococcus aureus*, and *Clostridium perfringens* affect one in six people annually, leading to approximately 128,000 hospitalizations and 3000 deaths, costing about USD 90 billion in the US. Many of these pathogens are on the global priority pathogen list of antibiotic-resistant bacteria provided by the National Institutes of Health (NIH) and the World Health Organization [1,6]

The anticipated doubling of the global demand for food and international trade in food over the next few decades is expected to drive a significant increase in foodborne diseases [7]. Climate change, the emergence of new pathogens and toxins, an increase in the number of at-risk (immunocompromised and aging) consumers, and shifting patterns of human consumption due to current preferences for fresh and minimally processed foods are additional factors that will pose significant challenges to global food safety [8].

Pathogenic microorganisms can easily taint food, becoming a stigmatized poison that could harm the consumer. Currently, 31 identified and countless unidentified pathogens have been reported as responsible for foodborne illness globally. Approximately 66% of diseases originate solely from pathogenic bacteria, and the association of these microorganisms in mono and mixed biofilms may increase bacterial intoxication and infection-related problems in humans [3]

Bacterial biofilms pose significant challenges to food safety and public health. These microbial communities adhere to surfaces, forming a protective matrix that enhances their resistance to antimicrobial agents and environmental stressors [9,10]. In the food industry, biofilms can lead to persistent contamination, foodborne illness outbreaks, and economic losses [9,11]. Common foodborne pathogens like *Listeria monocytogenes*, *Staphylococcus aureus*, *Salmonella enterica*, and *Escherichia coli* can form biofilms, with some bacterial molecules serving dual functions in biofilm formation and pathogenicity [10]. Biofilms also contribute to antibiotic resistance, complicating the treatment of infections [12]. Researchers are exploring various strategies to control and eliminate biofilms to address these issues, including improved cleaning and sanitization protocols [9,11]. The concept of “One Biofilm” has been proposed, emphasizing the interconnected nature of biofilms across humans, animals, and the environment. This idea aligns seamlessly with the “One Health” approach, which aims to address foodborne pathogens by integrating efforts across these sectors [13]. In 2017, the EU officially adopted the “One Health” approach to combat antibiotic resistance in both animal and human medicine and to prevent the transmission of zoonotic diseases [14]. A unified “One Health” approach is essential for managing food safety and understanding the drivers and determinants behind the emergence and persistence of biofilm-related challenges. Understanding biofilm formation mechanisms and their growth requirements is crucial for developing effective control methods and ensuring food safety [11,12].

### 1.2. Green Antimicrobial Agents as a Potential Solution

Green antimicrobial agents offer a promising alternative to conventional treatments for controlling bacterial biofilms, which are known for their resistance to traditional methods. These natural anti-biofilm agents, such as antimicrobial peptides, phytochemicals, biosurfactants, and weak organic acids, have demonstrated the ability to modulate biofilm formation and persistence [15]. Among these, green-synthesized nanoparticles, particularly silver nanoparticles, have gained attention due to their small size and capacity to penetrate microbial cells, exhibiting significant antimicrobial activities both independently and in combination with antibiotics [16]. These nanoparticles, synthesized using plant extracts and microbes, offer an environmentally friendly alternative, potentially addressing the challenge of multi-drug resistance [17]. Other green biocides, including essential oils, enzymes, and volatile organic compounds produced by bacteria, have shown potential in biofilm control, providing sustainable solutions across various applications [18].

Bacteriophages, viruses that specifically infect and lyse bacteria, are emerging as promising green antimicrobial agents to combat antibiotic-resistant pathogens [19,20]. These natural enemies of bacteria have demonstrated effectiveness against multidrug-resistant strains both in vitro and in vivo, offering a potential alternative to conventional antibiotics [19,21]. Phage therapy, with its historical roots in Eastern Europe and the former Soviet Union, is regaining interest globally due to the growing antibiotic resistance crisis [22,23]. In the context of food production, bacteriophages show promise as biocontrol agents, bio-sanitizers, and bio-preservatives against foodborne pathogens, aligning well with sustainable development goals [24]. Their specificity, natural occurrence, and compatibility with humans and animals make them attractive candidates for farm-to-fork applications [14]. However, further research is necessary to address challenges such as formulation, stability, and the potential for phage resistance [24].

Despite these advances, traditional control methods continue to face challenges due to bacterial adaptation, necessitating the exploration of alternative strategies [25]. Natural antimicrobials, such as bacteriocins produced by lactic acid bacteria, have shown efficacy in inhibiting biofilm formation, particularly against pathogens like *Listeria monocytogenes* and *Staphylococcus aureus* [26]. Moreover, phytogenic compounds have demonstrated effectiveness against *Vibrio parahaemolyticus* biofilms in seafood processing environments [27]. These green antimicrobial agents not only enhance food safety but also contribute to environmental sustainability. However, the eradication of preformed biofilms remains a significant challenge. Combining these agents with other antimicrobials or incorporating them into nanoconjugates may enhance their efficacy [26]. Future research should focus on developing multitargeted approaches and exploring antivirulence strategies to combat foodborne pathogen biofilms more effectively [25,27].

In summary, while green antimicrobial agents, including bacteriophages, present a promising avenue for biofilm control, addressing the complexity of biofilm-associated infections will require continued innovation and a multidisciplinary approach, aligning with the broader goals of the “One Health” framework. Understanding the interconnected nature of biofilms in humans, animals, and the environment is crucial for advancing these strategies and ensuring sustainable solutions for food safety [12,13,14,28].

## 2. Bacterial Biofilms

### 2.1. Definition and Characteristics of Bacterial Biofilms

Bacterial biofilms are complex communities of microorganisms attached to surfaces and encased in a self-produced extracellular matrix [29,30]. These sessile communities exhibit distinct characteristics compared to their planktonic counterparts, including increased antibiotic resistance, disinfectants, and host immune responses [29,31]. Biofilm formation is a dynamic process involving initial cell–surface interactions, maturation, and eventual dispersal [32]. Key features of biofilms include oxygen and nutrient gradients, reduced metabolic activity in deeper layers, and the presence of persister cells [29]. Biofilms are ubiquitous in the environment and are associated with over 60% of human infections [30,31].

### 2.2. Formation, Structure, and Functions of Biofilms in Food Environments

Biofilms pose significant challenges in food processing environments, as they can harbor pathogenic and spoilage microorganisms, making them more resistant to antimicrobial treatments and physical stresses [33]. The matrix protects bacteria from various environmental stressors, including UV radiation, extreme pH, and antibiotics [34,35]. Biofilms can form on food surfaces, processing equipment, and packaging materials, compromising food safety and sanitation [36,37,38]. Recent research has focused on developing biological and chemical methods for preventing biofilm formation and removing existing biofilms in food processing environments [35].

**Figure 1:**
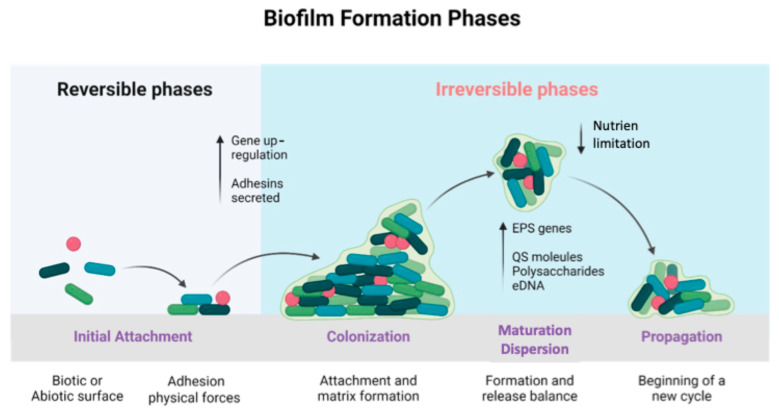
Biofilm formation phases. The following are four phases of biofilm formation: Initial Attachment: free living bacteria can temporarily connect to a suitable surface by using cell appendages, and then they can permanently attach by secreting adhesins. Colonization: is formed by the upregulation of genes necessary for maintaining attachment. Maturation–Dispersion: the development of the biofilm by the activation of EPS genes, and the release of polysaccharides, eDNA, and QS molecules. Finally, the dispersal of bacteria living in biofilm happens after a given amount of time, when nutrients are depleted and harmful chemicals start to build up. Then, the cycle begins again. Created with BioRender.com.

Biofilms in food environments are formed when bacteria and other microorganisms adhere to biotic or abiotic surfaces in the presence of moisture, nutrients, and suitable conditions [34]. Biofilm formation is a multifactorial and complex process that requires the activation of specific signaling pathways, such as quorum sensing, and the transcription of a set of genes distinct from those in planktonic forms of the same bacterial species [39,40]. The formation of biofilms follows a multi-stage process (Figure 1) involving reversible attachment of bacterial cells to a surface, followed by irreversible attachment, where cells adhere more firmly [41,42]. Subsequent stages include maturation, where microcolonies form and develop into larger aggregates encased in a self-produced matrix, and finally dispersion [41,43]. The matrix composition varies but often includes polysaccharides, proteins, and extracellular DNA [43]. Biofilm formation is regulated by genetic and environmental factors, with cyclic di-GMP playing a crucial role in many Gram-negative species [42]. Biofilms contribute to bacterial persistence and antibiotic resistance, making infections difficult to treat [43,44].

### 2.3. Challenges Posed by Bacterial Biofilms in Food Safety

Bacterial biofilms pose significant challenges to food safety by forming resilient communities on food processing surfaces, leading to cross-contamination and foodborne illnesses [9,12]. These biofilms are encased in extracellular polymeric substances, making them highly resistant to sanitizers and antibiotics, as this acts as a protective shield, safeguarding microorganisms against various forms of environmental stress, including desiccation, temperature fluctuations, and the presence of antimicrobial agents [12,45]. This matrix enables microorganisms to survive in unfavorable conditions, thereby promoting their persistence. Biofilm-associated microorganisms exhibit a higher resistance to antimicrobial agents compared to their planktonic counterparts. The biofilm’s architecture, combined with the extracellular matrix, impedes the infiltration of antimicrobial agents, reducing their efficacy in eradicating these microorganisms. Common foodborne pathogens like *Listeria monocytogenes, Staphylococcus aureus, Salmonella enterica,* and *Escherichia coli* form biofilms as a survival strategy, while *Pseudomonas aeruginosa* aids in the persistence of polymicrobial biofilms [10]. This persistence increases the risk of cross-contamination and the subsequent transmission of pathogens to food products. Furthermore, the detachment of microorganisms from biofilms during handling and processing stages can lead to food contamination. Biofilms often harbor diverse microbial species that engage in mutual interactions, influencing microorganisms’ viability and metabolic activity within the biofilm. These interactions can yield both positive and negative outcomes, further complicating efforts to control biofilm-related contamination.

The food industry faces significant economic losses due to biofilm-related contamination and equipment damage [45]. Effective control strategies require a thorough understanding of biofilm formation mechanisms, including surface attachment, environmental conditions, and bacterial cell interactions [9,45]. Enhanced cleaning and sanitization programs are essential to prevent biofilm formation and ensure food safety [9,45].

### 2.4. Antibiotic Resistance in Bacterial Biofilms

Bacterial infections pose a serious threat to human health, which has fueled extensive research into new antibacterial strategies. The development of antibiotics, starting with the discovery of penicillin by Sir Alexander Fleming in 1929, revolutionized the treatment of acute infections. However, this advancement also led to the emergence of slow-progressing persistent infections, often resistant to conventional antibiotic therapies [34]. In 1978, Costerton identified bacterial biofilms as the primary source of these chronic infections [46]. Today, it is recognized that about 65–80% of all infections are associated with biofilms, prompting researchers to deepen their understanding of these resilient bacterial communities and develop effective anti-biofilm strategies [47]. Bacterial biofilms are complex structures that exhibit increased antibiotic resistance compared to planktonic cells, posing significant challenges in clinical settings [48,49]. This multifactorial resistance involves both innate and induced mechanisms [50]. Key factors include reduced antibiotic penetration through the biofilm matrix, altered microenvironments leading to metabolic changes, formation of persister cells, and adaptive stress responses [48,49,50]. These mechanisms are not the result of mutations but rather the expression of wild-type genes in response to environmental stressors [49]. The biofilm’s extracellular polymeric matrix, composed of proteins, polysaccharides, and DNA, plays a crucial role in protecting bacteria from host defenses and antimicrobial agents [51].

Antimicrobial resistance (AMR) has become a critical public health issue worldwide, as infections caused by antibiotic-resistant pathogens complicate treatment, leading to increased morbidity and mortality. Over the past few decades, the misuse of antibiotics in both humans and food-producing animals has driven the emergence and spread of antibiotic-resistant bacteria [6]

To combat biofilm-related AMR, researchers have explored various natural anti-biofilm agents, including bacteriophages and plant extracts, which show promise in targeting biofilm and planktonic cells alike [52]. The protective nature of biofilms and the associated multidrug resistance pose significant challenges, but ongoing research is identifying natural compounds that may prevent biofilm formation and disrupt existing biofilms.

### 2.5. Mechanisms of Antibiotic Resistance in Biofilms

Bacterial biofilms exhibit enhanced antibiotic resistance through multiple mechanisms. These include reduced antibiotic penetration due to the extracellular matrix, decreased growth rates, and altered metabolic activity in nutrient-limited environments [32,50]. Biofilms also form persister cells, which are highly tolerant to antibiotics [53]. Quorum sensing plays a role in biofilm formation and resistance [54]. Specific genetic determinants contribute to both innate and induced resistance pathways [50]. Other mechanisms include enzyme-mediated resistance, efflux pumps, and genetic adaptations [54]. The combination of these defenses makes biofilm infections extremely difficult to treat [53]. Understanding these mechanisms is crucial for developing new therapeutic strategies, such as targeting quorum sensing or other biofilm-specific processes [32,50,55].

#### Key Mechanisms of Antibiotic Resistance in Biofilms

The mechanisms of resistance of bacterial biofilms differ from those of planktonic cells. They include the following:

*Physical Barrier:* Biofilms are complex microbial communities that adhere to surfaces, forming a protective matrix of biomolecules [56,57]. The physical properties of biofilms, including surface adhesion, viscoelasticity, and mechanical stability, play crucial roles in their development and resistance to environmental challenges [56,58]. These properties are influenced by various factors, such as cell motility, cellular adhesion, and the composition of extracellular polymeric substances [56,59]. Specific matrix components, like the surface-layer protein BslA in Bacillus subtilis NCIB 3610 biofilms, significantly affect surface roughness and elasticity [59]. The presence of γ-polyglutamate in B. subtilis B-1 biofilms protects against ethanol-induced changes in biofilm stiffness [59].

*Slower Growth Rate:* Bacteria within biofilms often exhibit a slower growth rate compared to planktonic bacteria. This reduced metabolic activity can make them less susceptible to antibiotics that target actively growing cells. Biofilms exhibit spatial variations in growth rates, with faster growth occurring at interfaces and slower growth in interior regions [60]. The rpoS gene plays a crucial role in biofilm formation and structure, with its deletion resulting in reduced biofilm cell density and altered cell arrangement [61]. The spatial structure and reduced growth rates in biofilms contribute to the emergence and maintenance of genetic diversity by limiting competition to a local scale and slowing the rate at which selection can alter genotype frequencies [62].

*Altered gene expression:* Biofilm bacteria can undergo genetic changes that lead to altered gene expression patterns. This can result in the upregulation of genes associated with antibiotic resistance mechanisms, such as efflux pumps that actively pump out antibiotics from the cell. Research on gene expression in bacterial biofilms reveals significant changes compared to planktonic growth. In *Escherichia coli* biofilms, genes involved in adhesion, auto-aggregation, and stress responses are upregulated, while many differentially expressed genes have unknown functions [63]. Similar patterns are observed in *Staphylococcus aureus*, with increased expression of cell envelope-related genes and stress responses in biofilms [64]. *Streptococcus mutans* biofilms show differential expression of about 12% of genes, with changes in expression patterns as biofilm thickness increases [65]. Environmental factors within biofilms, such as higher osmolarity, oxygen limitation, and increased cell density, contribute to these gene expression changes [66].

*Persister cells:* These are phenotypic variants within bacterial and fungal biofilms that exhibit high tolerance to antimicrobial agents, contributing to the recalcitrance of biofilm-associated infections [67,68]. These cells represent a small subpopulation in a dormant state, similar to glucose-deprived cells, and can be observed immediately after adhesion to a substrate [68]. These cells have reduced metabolic activity and are highly tolerant to antibiotics. They can serve as a reservoir for re-establishing the biofilm population after antibiotic treatment [69]. Mathematical modeling suggests that persisters accumulate in substrate-limited regions of biofilms and can quickly revert to normal cells after antibiotic treatment, allowing biofilm regrowth [70]. Persister formation is influenced by substrate type and stress response pathways, with cells accumulating stress-protecting molecules like glycogen and trehalose [68].

*Phenotypic heterogeneity:* Phenotypic heterogeneity in biofilms is a complex phenomenon arising from various factors. It can develop as an adaptive response to surface heterogeneity, with initial colonizers sensing different adhesion forces and formulating local responses [71]. This heterogeneity can include variations in antibiotic susceptibility, with some subpopulations exhibiting higher levels of resistance than others. This diversity helps protect the biofilm community as a whole. This heterogeneity can be influenced by the presence of cheaters, leading to increased phenotypic variation in matrix production or uniform adaptation strategies [72]. Within biofilms, bacteria undergo genomic and proteomic changes, resulting in enhanced antimicrobial tolerance, particularly through the formation of persister cells [73]. Two forms of cell heterogeneity coexist in biofilms: physiological responses to resource gradients across the biofilm structure, and local phenotypic variations due to stochastic gene expression [74].

*Quorum sensing:* Quorum sensing (QS) is a cell-density-dependent communication mechanism used by bacteria to regulate various behaviors, including virulence and biofilm formation [75,76]. This cell-to-cell communication allows them to coordinate their behavior and adapt to environmental changes. This process involves signaling molecules such as acyl-homoserine lactones (AHLs) and autoinducing peptides (AIPs) [77]. Inhibition of QS, known as quorum quenching, has emerged as a promising strategy to control bacterial infections and combat antibiotic resistance [75,77]. Quorum sensing inhibitors (QSI) primarily act at early stages of biofilm formation by interrupting bacterial communication and gene regulation for adhesion and EPS synthesis [78]. QS inhibitors can be classified into three main categories: nonpeptide small molecules, peptides, and proteins [76]. These inhibitors work by repressing signal generation, blocking signal receptors, or disrupting QS signals [76,79]. In *Pseudomonas aeruginosa*, QS inhibition can target the *Las* and *Rhl* systems, which control virulence factor expression [79]. QS inhibitors can be obtained from natural sources or synthesized in laboratories [79].

These mechanisms work together to enhance the survival and persistence of bacteria within biofilms, making them highly resistant to antibiotics. Understanding these mechanisms is crucial for developing effective strategies to combat biofilm-related infections and overcome antibiotic resistance.

## 3. Novel Strategies and Mechanisms of Action: Green Antimicrobial Agents

Novel strategies for combating bacterial biofilms and antibiotic resistance include the use of green antimicrobial agents or unconventional antimicrobials. These agents may have different mechanisms of action or be of natural origin, making them potential alternatives to conventional antibiotics.

### 3.1. Definition and Types of Green Antimicrobial Agents

Green antimicrobial agents are emerging as vital alternatives to combat multidrug-resistant pathogens. These agents refer to a category of substances that are derived from natural sources or designed to be environmentally sustainable. These agents exhibit antimicrobial properties and serve the purpose of regulating the proliferation and behavior of microorganisms, such as bacteria, viruses, and fungi [18]. The aforementioned agents are sourced from renewable resources and are widely regarded as more sustainable and environmentally friendly substitutes for conventional antimicrobial compounds, such as synthetic chemicals or antibiotics [80].

Green antimicrobial agents encompass a diverse range of natural substances, including essential oils, plant extracts, antimicrobial peptides, enzymes, and other bioactive compounds sourced from plants, animals, or microorganisms (Table 1) [19,81,82]. These agents frequently demonstrate wide-ranging antimicrobial activity, thereby possessing the ability to effectively target a diverse array of microorganisms. These agents have the potential to exert their effects through various mechanisms, such as the disruption of cellular membranes, interference with crucial cellular processes, or inhibition of microorganism growth and reproduction [83,84,85,86].

The utilization of environmentally friendly antimicrobial agents is increasingly recognized as a viable strategy in a range of fields, such as food safety, healthcare, agriculture, and the environment.

### 3.2. Bacteriophages as Antimicrobial Agents

Bacteriophages, also known as phages, are viruses that infect and kill bacteria. Bacteriophages have emerged as promising biocontrol agents against foodborne pathogens, offering an alternative to conventional methods and addressing concerns about antibiotic resistance [93,94]. These viruses can be applied at various stages of food production, including pre-harvest, post-harvest, and food processing environments, to control pathogens such as *Salmonella*, *E. coli* O157:H7, *Listeria monocytogenes*, and *Campylobacter* [95,96]. Phages also target mature biofilms by infecting bacteria embedded within the matrix and may produce enzymes that degrade EPS components, facilitating deeper penetration and biofilm collapse [34]. Phage-derived endolysins and cell-wall binding domains have shown potential for rapid food pathogen detection [94]. Several phage-based products have been approved by regulatory authorities for use in food safety applications [97]. While bacteriophages offer advantages such as high host specificity and natural occurrence in foods, challenges including phage resistance development need to be addressed [96]. Ethical concerns surrounding phage applications include the emergence of phage-resistant bacterial strains, potential gene transfer, and limited consumer acceptance due to the viral nature of these agents [98,99]. Regulatory and equity issues also arise, especially regarding the standardization of phage production and public access to phage-based treatments [100]. Addressing these concerns through genomic screening, public education, and controlled clinical trials will be essential for the safe and ethical deployment of bacteriophage strategies in food and healthcare settings.

Overall, bacteriophages represent a promising approach to enhance food safety and reduce foodborne illnesses.

#### 3.2.1. Mechanisms of Bacteriophages in Biofilm Control

Bacteriophages offer a promising alternative for controlling bacterial biofilms, which are resistant to conventional antibiotics and disinfectants [101]. Phages can effectively penetrate biofilms due to their small size and ability to diffuse through bacterial cell walls [102]. They employ various mechanisms to combat biofilms, including relocation through water channels, local replication, and the production of depolymerizing enzymes that break down the extracellular polymeric matrix [102,103]. Phage-derived enzymes, such as endolysins and depolymerases, play a crucial role in biofilm eradication [102]. The use of phage cocktails can prevent the development of phage-resistant bacteria [103]. Bacteriophages, often combined with other agents like antimicrobial peptides, show potential in treating biofilm infections caused by various pathogens, including ESKAPE organisms [83]. This approach offers a safe and effective alternative to conventional treatments in medical, industrial, and environmental applications [101].

#### 3.2.2. Phage-Derived Enzymes as Antibiofilm Agents and Role in Biofilm Degradation

Bacteriophages and their enzymes have emerged as promising agents for biofilm control and treatment of bacterial infections. Phage-derived enzymes, particularly peptidoglycan hydrolases (lysins) and polysaccharide depolymerase, can effectively degrade biofilm structures and kill bacteria, including antibiotic-resistant and persister cells [104,105]. These enzybiotics demonstrate high efficiency, pathogen specificity, and low potential for resistance development [104]. Enzymes that degrade biofilm matrix polymers, such as deoxyribonuclease I and dispersin B, have shown promise in inhibiting biofilm formation, detaching established colonies, and increasing bacterial susceptibility to antimicrobial agents [106]. Additionally, engineered bacteriophages producing quorum-quenching enzymes, like lactonase, can inhibit biofilm formation by disrupting bacterial cell–cell communication [107]. While phages and phage-derived products have shown success in destroying biofilms, they are often insufficient to eradicate all bacterial cells when used alone [108]. Combining phages or phage-derived enzymes with other antimicrobials, such as antibiotics, nanoparticles, or antimicrobial peptides, may enhance their effectiveness in removing biofilms and treating associated infections [108,109]. These phage-based strategies have demonstrated efficacy in various animal models and are now entering clinical trials, offering promising alternatives to combat the global threat of antibiotic-resistant bacteria [104,110].

### 3.3. Plant Extracts: Essential Oils as Potential Antimicrobial Agents

Essential oils (EOs) are emerging as potential alternatives to synthetic antimicrobial agents due to their effectiveness against drug-resistant pathogens [111]. These have gained attention as natural antimicrobial agents due to their complex bioactive nature and potential to combat foodborne pathogens and spoilage organisms [112]. Their antimicrobial mechanisms involve targeting multiple cellular components and processes, including cell membranes, enzymes, and metabolic pathways [113]. EOs exhibit a wide spectrum of activity against bacteria, fungi, viruses, and parasites [114]. These complex volatile compounds, derived from medicinal and aromatic plants, contain various antimicrobial components such as aldehydes, phenolics, and terpenes [115].

Their antimicrobial properties make them valuable in medicine, agriculture, and cosmetology [116]. Additionally, EOs show promise as food preservatives for cereals, grains, pulses, fruits, and vegetables due to their natural, safe, and biodegradable nature [117]. Studies have demonstrated the potent antimicrobial properties of both liquid EOs and their volatile constituents, with rosemary, tea tree, and cassia showing particularly strong broad-spectrum antibacterial activity [118]. While numerous EOs possess antimicrobial potential, only a select few are highly effective [116]. Despite their promise, EOs face challenges in commercial applications due to strong flavors, volatility, and chemical instability. To address these limitations, food-grade delivery systems are being developed to improve the efficacy of EOs as natural antimicrobials in food products [112].

EOs exhibit multiple mechanisms of action, including destruction of cytoplasm membranes and inhibition of protein synthesis [111]. Recent advances in genomics and proteomics have enhanced our understanding of their mode of action [119]. Nanoencapsulation of EOs has shown promise in improving their chemical stability, solubility, and efficacy against multidrug-resistant pathogens [120]. Additionally, synergistic combinations of EOs with other antimicrobial compounds have been explored to overcome antibiotic resistance [120]. However, further research is needed to elucidate the specific mechanisms of action for individual EO components and their interactions with food matrix components [112,120]. However, it is important to note that the antimicrobial activities of EOs are not universal for all microbial strains, necessitating further research to target specific EOs and microorganisms [116,121]. Overall, EOs offer a promising avenue for combating antimicrobial resistance and preserving food products.

Additionally, EOs and their major components have demonstrated significant antibiofilm activities against various pathogens. Studies have shown that tea tree oil, α-terpineol, terpinen-4-ol, and Melissa officinalis EO exhibit stronger antibiofilm effects compared to lavender EO and its constituents [122]. Cinnamon and palmarosa oils have been found to completely inhibit *Escherichia coli* planktonic cells, while green tea EO and terpinen-4-ol showed fewer effective results against biofilms [123]. Among six tested EOs, cinnamon and oregano oils demonstrated the strongest antimicrobial and antibiofilm activities, followed by citronella, rosemary, and eucalyptus oils with moderate effects [124]. EOs generally showed better antifungal activity compared to antibacterial effects, with stronger action against Gram-positive cocci than Gram-negative bacilli [124]. Several EOs can modulate gene expression involved in biofilm formation and virulence factors [125]. Interestingly, enantiomeric monoterpenes affect quorum sensing regulation differently, with (+)-enantiomers increasing violacein formation and (−)-enantiomers inhibiting it [125]. Essential oils and their components show potential as effective alternatives to conventional antimicrobials for controlling biofilms in food and healthcare industries [123,125]. These findings suggest that EOs and their components have potential applications in developing new antimicrobial and antibiofilm products.

### 3.4. Combining Green Antimicrobial Agents for Biofilm Control 

Combining antimicrobial agents with essential oils (EOs) shows promise in combating drug-resistant foodborne pathogens. EOs exhibit multi-target inhibitory effects on microorganisms, enhancing the activity of traditional antimicrobials and potentially preventing the emergence of resistance [126]. Synergistic interactions between EOs and antimicrobials have been observed against various pathogens, including *Salmonella enterica*, with significant reductions in minimum inhibitory concentrations for both components [127]. This approach addresses the need for more effective antimicrobial strategies in food processing, offering enhanced inactivation of pathogens while minimizing the impact on food quality [128]. Developing such combined treatments is crucial as antimicrobial resistance in foodborne microorganisms poses a significant public health hazard [129]. Further research is needed to elucidate the chemical and microbiological mechanisms underlying these synergistic effects and to overcome challenges in industrial implementation [129,130,131]. Bacteriophages and essential oils (EOs) show promise as natural biocontrol agents against foodborne pathogens. Studies have demonstrated their effectiveness in reducing *Salmonella enteritidis* biofilms on food-contact surfaces and eggshells [132] inactivating *Escherichia coli* O157:H7 on leafy greens [133]. The combination of phages and EO compounds has proven more effective than individual treatments in reducing bacterial contamination and gene expression [132]. Phages and phage-derived endolysins have been successfully applied to control various foodborne pathogens in foods, offering potential alternatives to conventional preservatives [94]. Additionally, phage-based detection systems have been developed for rapid and specific identification of viable pathogens in foods [94]. The increasing incidence of foodborne illnesses and antibiotic-resistant bacteria has further motivated research into phage applications for pre- and post-harvest interventions in the food industry [95].

Biofilms formed by foodborne pathogens pose significant challenges to food safety and industry. Conventional sanitizers often struggle to eradicate these resilient structures, necessitating novel approaches. Combining treatments has shown promise, such as pairing chemical sanitizers with steam heating for enhanced biofilm reduction [134]. Antimicrobial peptides (AMPs) from lactic acid bacteria offer a natural alternative, effectively inhibiting biofilm formation in a dose-dependent manner [26]. AMPs can be further potentiated when combined with antibiotics, matrix-disaggregating compounds, or quorum-sensing inhibitors [135]. Other innovative strategies include surface modifications, cell-signaling inhibition, enzymatic disruption, and the use of bacteriophages, bacteriocins, and plant essential oils [36]. These combination approaches target different aspects of biofilm formation and maintenance, potentially offering more comprehensive control of foodborne pathogens in industrial settings (Table 2).

While green antimicrobial agents offer promising eco-friendly alternatives, several limitations challenge their widespread application. These include chemical instability under industrial processing conditions [100], compositional variability among natural extracts [142], and lack of standardization in formulations [143]. In addition, some agents may induce undesirable sensory changes or pose toxicity risks at higher concentrations [144]. Regulatory hurdles and limited public familiarity, particularly with bacteriophages, further restrict their immediate implementation in food systems [141].

## 4. Conclusions

This review has highlighted the mechanisms and applications of bacteriophages, plant-derived compounds, and phage-associated enzymes as some of the most synergistic and targeted options currently under investigation.

When using combined green antimicrobials, also called synergistic antimicrobial combinations, two or more natural or environmentally friendly antimicrobial agents are used together or one after the other to make them more effective against pathogens. Antimicrobials derived from natural sources, such as plant extracts, essential oils, bacteriophages, and phage-derived enzymes, are considered eco-friendly alternatives to conventional antimicrobials.

The use of combined green antimicrobials has several possible benefits. First, it may broaden the spectrum of antimicrobial activity, as different green antimicrobial agents may have varying mechanisms of action and target different microorganisms. This can result in a synergistic effect where the combined antimicrobial agents exhibit enhanced activity against a wider range of pathogens than individual agents alone.

Second, the use of combined green antimicrobials may help overcome the issue of antimicrobial resistance. By utilizing multiple antimicrobial agents with different mechanisms of action, the development of resistance by microorganisms may be mitigated, as it would require simultaneous resistance to various agents, which is less likely to occur. While these natural agents offer eco-friendly, biodegradable, and selective antimicrobial action, several challenges must still be addressed to enable their large-scale adoption. These include their physicochemical instability, variability in efficacy due to natural origin, sensory limitations in food systems, and regulatory hurdles. Moreover, the potential development of resistance, especially in the case of bacteriophages, raises important ethical and safety considerations that must be addressed through genomic screening, public education, and controlled trials.

Future research should focus on enhancing the stability and delivery of green antimicrobial agents through advanced encapsulation systems and material integration. Investigating synergistic combinations in more complex, real-world matrices such as food surfaces, packaging films, and wound biofilms will be crucial for understanding their practical potential. Moreover, standardized efficacy testing protocols, predictive modeling of biofilm interactions, and safety evaluations will be essential to advance their commercial and clinical adoption.

Expanding knowledge about antimicrobial peptides, biosurfactants, and the bioactivity of plant waste derivatives could further strengthen the portfolio of green interventions. Ultimately, the integration of multi-targeted, safe, and sustainable antimicrobial strategies holds great potential for biofilm control in food safety and public health.

## Figures and Tables

**Table 1:** Types and mechanisms of green antimicrobial agents.

Type of Agent	Main Mechanism	Typical Application	Example–Reference
Essential Oils	Membrane disruption, QS inhibition	Food preservation, surfaces	Carvacrol, eugenol [87]
Plant Extracts	Polyphenols, antioxidants, antimicrobial action	In vitro biofilms, packaging	Green tea, grape, rosemary [88]
Antimicrobial Peptides (AMPs)	Membrane interaction → pore formation or lysis	Combined with enzymes or nanoparticles	Nisin, defensins, bacteriocins [89]
Bacteriophages	Specific bacterial lysis; endolysin release	Food surfaces, infections	Listeria in deli meats [6]
Enzymes (e.g., endolysins, dispersins)	EPS matrix degradation	Combined with phages or EO	Dispersin B + phage [90]
Green Nanoparticles	Targeted delivery + catalytic activity	Encapsulated formulations	AgNPs with EO [91]
Microbial Biosurfactants	Surface tension reduction; antiadhesion	Biofilms, formulations	Rhamnolipids [92]

Overview of common green antimicrobial agents, their mechanisms of action, and examples of application in food and biofilm-related contexts.

**Table 2:** Synergistic applications of green antimicrobial agents.

Combination	Target Bacteria	Application Matrix	Synergistic Effect
Phage + Honey [136]	*E. coli*	In vitro biofilm	Enhanced killing vs. phage alone
Phage + Endolysin [137]	*L. monocytogenes*	Food-contact surface	Biofilm disruption > 70%
Phages + Cinnamaldehyde [138]	*E. coli*, *Salmonella*	Alginate-based films	Significant CFU reduction
EO + Phage endolysin [139]	*Salmonella Typhimurium*	Cooked ground beef	Increased antimicrobial activity
Phages + Enzymes + EO [140]	Multiple pathogens	Active packaging	EPS matrix degradation + phage delivery
Phages+ Enzybiotics [141]	*Listeria, Salmonella*	Food biofilms	Specificity + matrix penetration

Representative studies demonstrating the synergistic effect of green antimicrobial agents—particularly phages, enzymes, and essential oils—against foodborne pathogens in biofilm or food-related matrices.

## Data Availability

No new data were created or analyzed in this study. Data sharing is not applicable to this article.
